# A Case of Redundant Sigmoid Colon and Sigmoid Volvulus

**DOI:** 10.7759/cureus.60508

**Published:** 2024-05-17

**Authors:** Kesav Sudabattula, Anup Zade, Darshana Tote, Srinivasa Reddy, Tejaswini Panchagnula, Tushar Dahmiwal

**Affiliations:** 1 General Surgery, Jawaharlal Nehru Medical College, Datta Meghe Institute of Higher Education and Research, Wardha, IND; 2 General Surgery, Mahatma Gandhi Institute of Medical Sciences, Wardha, IND; 3 General Surgery, Sapthagiri Institute of Medical Sciences and Research Centre, Bengaluru, IND

**Keywords:** sigmoid volvulus, sigmoidectomy, peritonitis, intestinal obstruction, abdominal distension

## Abstract

The torsion of a dilated sigmoid colon around its own mesenteric axis is the cause of sigmoid volvulus, which frequently results in constipation and intestinal obstruction. The clinical presentation of sigmoid volvulus can be observed as nausea, constipation, abdominal distension, and abdominal pain. It is also reported to be insidious. Additionally, it causes blood obstruction, resulting in necrosis, bowel ischemia, and even intestinal perforation if not addressed on time. Physical symptoms might vary depending on the course of the disease but are usually observed as the classical trio of abdominal distension, abdominal pain, and constipation. Computed tomography imaging presents the sign of an inverted U, or classic coffee bean, aiding in the diagnosis of the sigmoid volvulus. A 38-year-old male was admitted to the emergency department of our tertiary care center with significant complaints of obstipation and abdominal pain. The medical history and physical examination revealed peritoneal symptoms, which warranted a prompt radiological imaging diagnosis. The patient was subjected to computed tomography, which was suggestive of sigmoid volvulus. The patient underwent an emergency laparotomy and sigmoidectomy, which were uneventful with no postoperative complications.

## Introduction

Colonic volvulus, which primarily affects the sigmoid colon and cecum, is globally the third most common cause of colonic blockage [1]. The sigmoid colon coils around its mesentery to form the sigmoid volvulus, resulting in a closed-loop blockage. If untreated, it can lead to potentially fatal consequences, including gangrene, perforation, and intestinal ischemia. Adults are typically affected by sigmoid volvulus, with the highest incidence observed between 40 and 80 years of age [2]. Some geographical regions have also been termed "volvulus belts" due to the high incidence rates of sigmoid volvulus, which majorly include countries such as India, the Middle East, Russia, Pakistan, Scandinavia, Eastern Europe, Africa, and Latin America. There is a varied range of incidence of sigmoid volvulus, with 3% on the lower end and going as high as 54% of total acute presentations of intestinal obstructions [3]. Acute sigmoid torsion, repeated prior torsion, or ileo-sigmoid knotting can be counted as possible presentations of sigmoid volvulus [4]. Diagnosis can be made by physical examination, plain abdomen radiography, barium enema, endoscopic tests, magnetic resonance imaging (MRI), and computed tomography (CT) scans. The CT scan is considered the gold standard and a crucial diagnostic measure to detect associated issues [4,5]. Management of sigmoid volvulus is critical and attributed primarily to acute presentation, irrespective of the location, comorbidities of the patient, or viability of the colonic wall. Though a significant fatality and morbidity rate has been associated with emergency surgery, it is required if there are clinical or radiological indicators of gravity. An endoscopic detorsion approach is the optimum line of action for sigmoid volvulus, followed by a sigmoid colectomy with primary anastomosis within two to five days if required [1-5]. We present the case of a patient who was operated on for resection and anastomosis of the redundant sigmoid colon, which had undergone sigmoid volvulus.

## Case presentation

A 38-year-old male was admitted to our tertiary care center in central India due to nonspecific abdominal pain and obstipation for three days. The patient did not experience nausea, headaches, or vomiting. There was no significant medical history noted. Additionally, there was no history of any surgical interventions. The patient appeared ill, and upon admission into the emergency room, his vital values were 120/80 mmHg blood pressure and 90 beats/minute. A physical examination showed the abdomen was diffusely painful and distended without guarding or rebound tenderness. All other examinations were found to be normal. The laboratory investigations are detailed in Table 1.

**Table 1:** Blood investigations of the patient g/dL: grams per deciliter, µL: microliter, mmol/L: millimoles per liter, mg/dL: milligrams per deciliter

Parameter	Patient Values	Normal Range
Hemoglobin	14.1 g/dL	13-17 g/dL
White blood cell count	5.6 thousand/µL	5.6 thousand/µL
Serum potassium	4.5 mmol/L	3.5-5.1 mmol/L
Serum sodium	140 mmol/L	135-145 mmol/L
Urea	19 mg/dL	19-43 mg/dL
Creatinine	0.7 mmol/L	0.66-1.25 mg/dL

**Figure 1:**
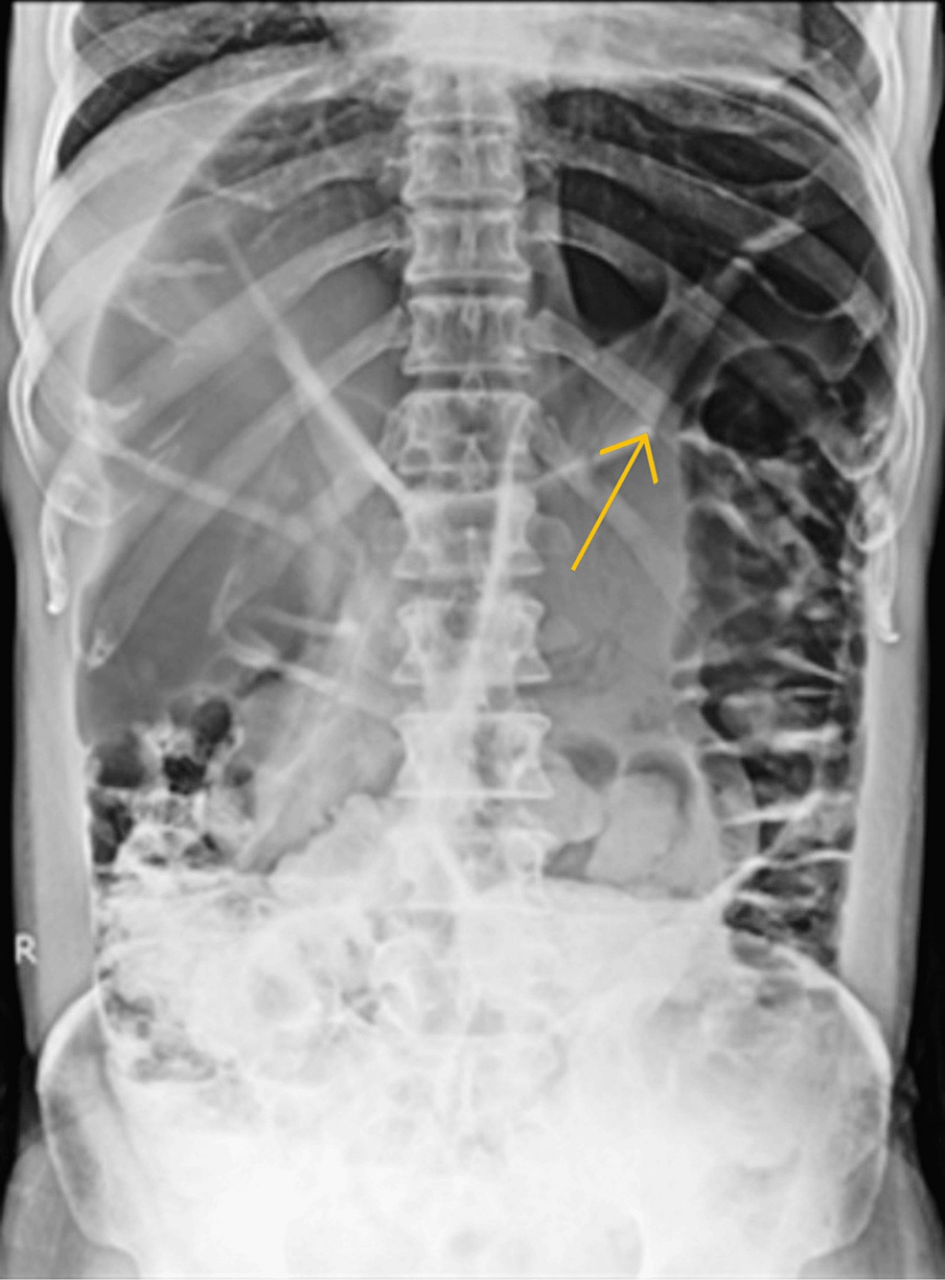
X-ray revealing a dilated sigmoid loop with a classic coffee bean sign The arrow shows the coffee bean sign.

A dilated sigmoid loop with a classic coffee bean sign, or the omega loop, was visible on the abdomen in the upright, supine position of the patient. A chest X-ray of the upright plain film revealed no sub-diaphragmatic free air (Figure 1).

**Figure 2:**
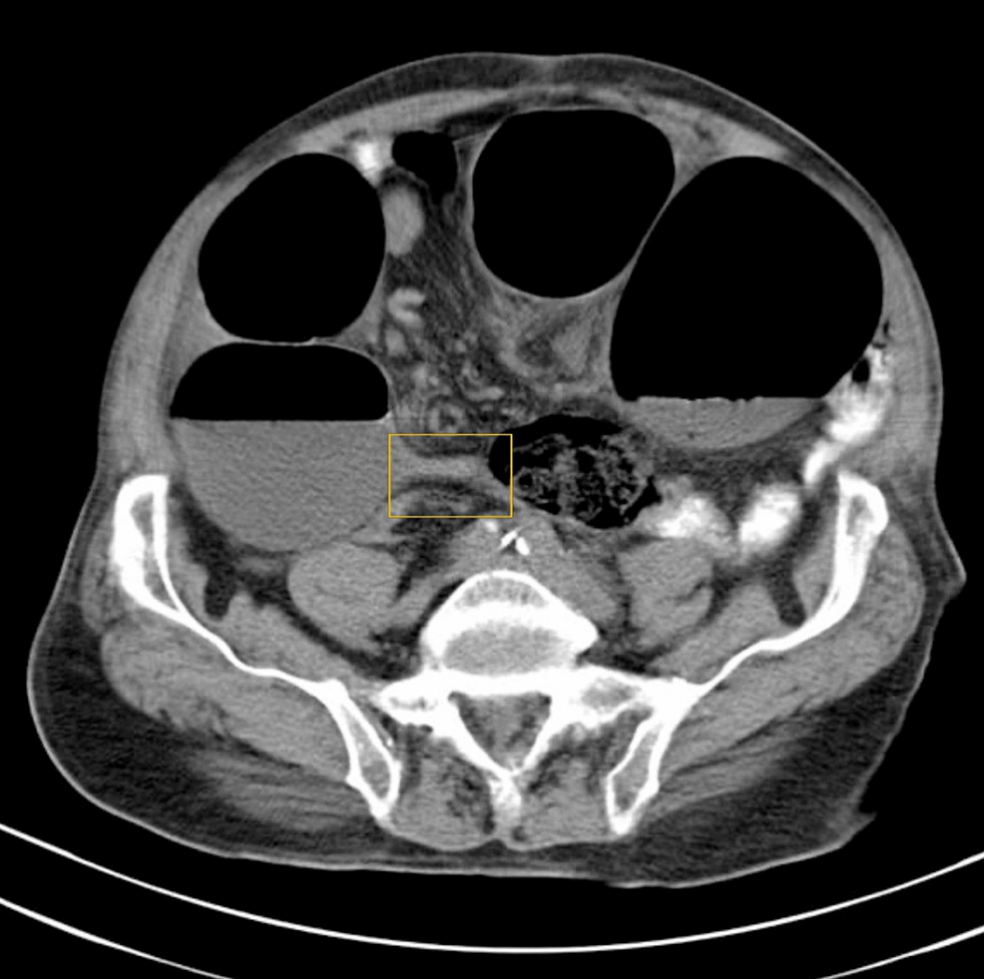
Computed tomography image of the patient The highlighted area indicates the transition point of the sigmoid volvulus.

A computed tomography scan was carried out for the confirmation of the diagnosis (Figure 2).

**Figure 3:**
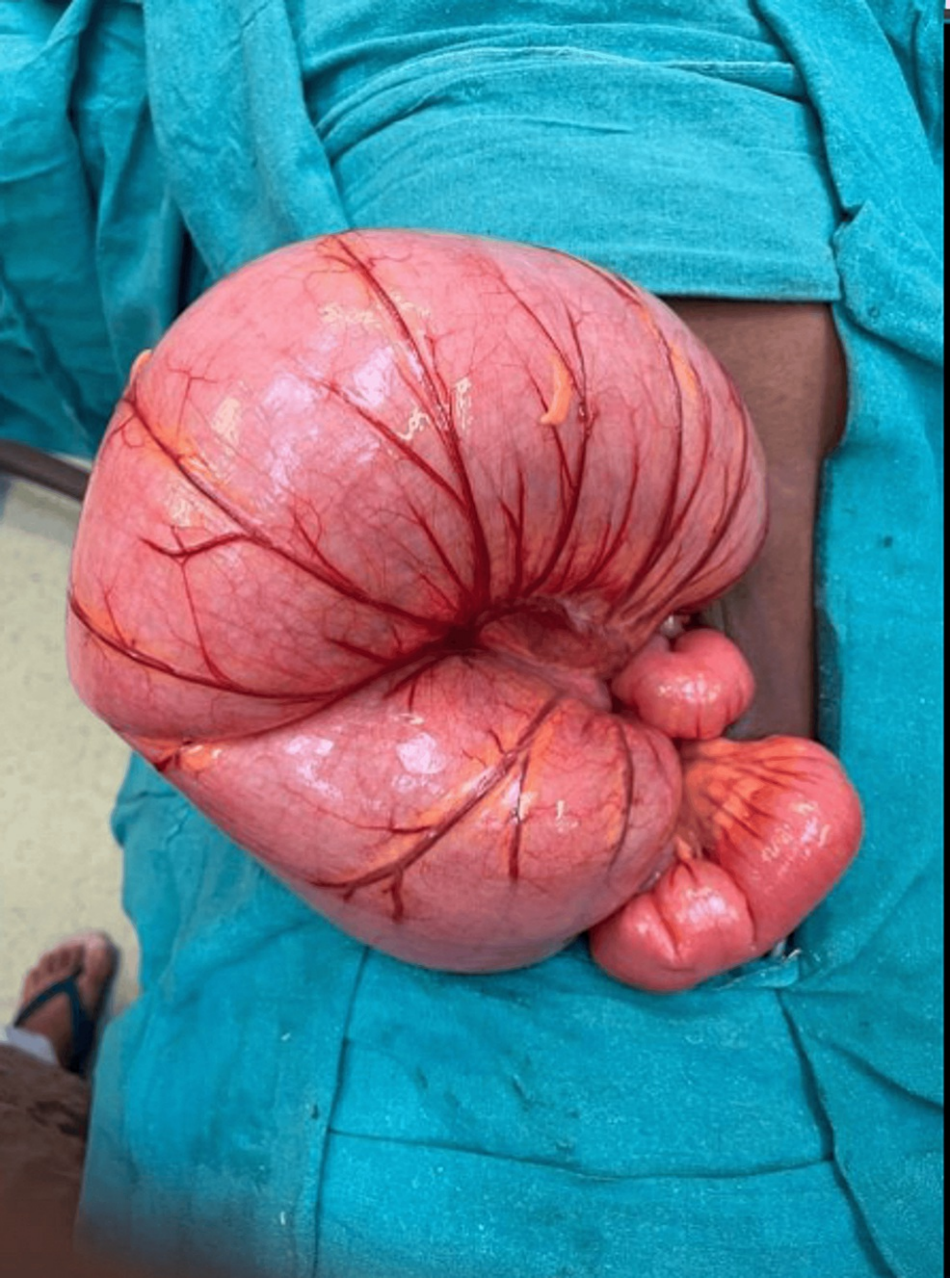
Dilated sigmoid colon

**Figure 4:**
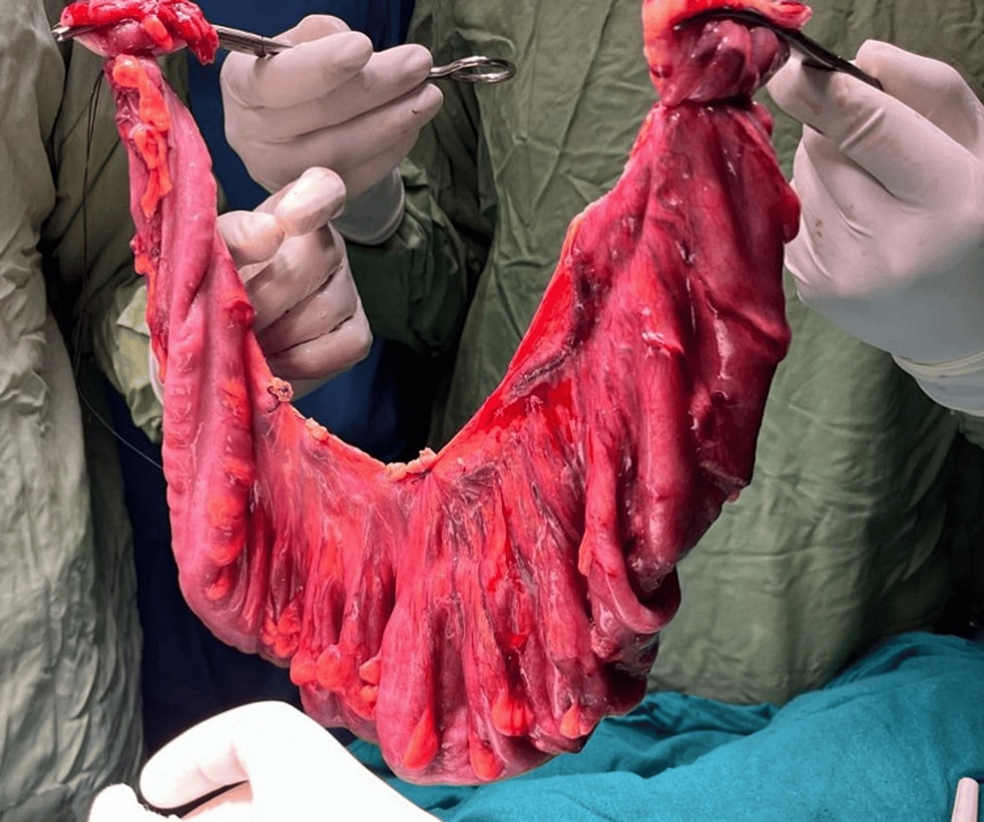
Excised volvulated segment specimen

Following an urgent laparotomy, it was determined that the patient had a sigmoid volvulus, which was not gangrenous. The patient underwent resection and anastomosis of the redundant sigmoid colon, which had undergone sigmoid volvulus. The primary anastomosis was performed, and an intra-abdominal drain was kept in the pelvic gutter. Postoperatively, the patient was mobilized on day one, and per rectal stimulation was given twice daily and started on a standard antibiotic regimen for broad spectrum. The patient started passing flatus on day two with sluggish bowel sounds heard. Thereafter, a liquid diet was initiated, followed by a soft diet. On postoperative day four, the patient passed stools. During the course of the hospital stay, only a minimal amount of drainage was observed, which was eventually removed on day five. The patient was discharged on day six. The patient was monitored twice daily during the course of her stay. The excised specimen of the dilated sigmoid colon is shown in Figures 3, 4.

The patient was followed up postoperative day 12 with a good recovery.

## Discussion

Colonic volvulus is a clinical condition commonly causing colonic blockage by affecting the cecum and sigmoid colon [1,2]. Publications report that the large bowel can develop volvulus in any part of it. Adults are typically affected by sigmoid volvulus between their fourth and eighth decades of life [2,5]. Though it was observed commonly in adults, currently, there are case reports from different parts of the world with its incidence across all age groups [6,7]. Sigmoid volvulus is clinically presented with symptoms such as abdominal distention, progressive pain in the abdomen, and nausea, and if not timely managed, can result in the development of severe complications such as intestinal ischemia, peritonitis, sepsis, and even mortality. A few cases like ours need to be operated on in an emergency due to the acute onset of the disease. Emergency clinicians and radiologists need to be vigilant regarding the obvious and subtle signs from imaging to enable a timely sigmoid volvulus. The mortality rates in patients with severe intestinal ischemia have been reported to range from 11% to 80%, while for patients with non-severe intestinal ischemia, the reported mortality rates range from 6% to 24% [8,9]. Cecal volvulus affects younger women, while sigmoid volvulus primarily affects elderly males. Several risk factors are similar in both locations, including aging, neuropsychiatric disorders, anatomic variations, megacolon, chronic constipation, Chagas disease, Hirschsprung disease, scleroderma, anatomic predispositions, and previous abdominal surgery [1,2].

The most significant predisposing factor for volvulus is considered to be a lengthy, mobile colonic segment with a limited mesenteric base. If left untreated, it can result in life-threatening consequences such as gangrene, perforation, and intestinal ischemia [1,9]. Based on clinical course, sigmoid volvulus has been defined into two groups. A total blockage manifesting clinically as abrupt-onset pain in the peri-umbilical region accompanied by emesis and constipation causes acute fulminating volvulus. Examining a patient often reveals peritoneal signs. The most commonly observed early consequences of this kind of volvulus are gangrene and perforation. In contrast, patients with subacute progressive volvulus have a more subtle beginning due to partial occlusion [3-5]. A more subdued clinical picture characterizes the sub-acute variant of sigmoid volvulus. It is frequently worse on the left side and is more commonly observed in older adults. Delays in diagnosis are often caused by the clinical signs of subacute progressive volvulus being overstated [2].

The management of sigmoid volvulus involves surgical and nonsurgical resections on the basis of disease severity, which is usually a decompression followed by a sigmoid resection by an open or laparoscopic approach [10]. Nevertheless, following a successful endoscopy, recurrence is reported in approximately 25% of patients, and some reports are as high as two-thirds of the total treated cases. Surgical resection might be recommended in cases with acute presentations similar to this case, where no evidence of bowel edema or gangrene at the resected margins was noted, and the surgeons can be more confident of the anastomosis performed.

Preventative treatment in the form of primary anastomosis and sigmoid resection should be taken into consideration, as this has also been demonstrated to be relatively safe in low-risk patients (ASA score <3) [11]. An endoscopic approach is recommended for patients who pose an unacceptable risk for surgery [1,10]. Clinical and radiological data are typically used to establish the diagnosis of acute sigmoid volvulus. Abdominal pain and distension, partial or complete constipation, tympanic abdomen, abrupt bowel sounds, and abnormal abdominal mass can be counted as early prognostic indicators accompanied by a conventional X-ray confirming the diagnosis in the majority of the patients [11]. This can aid the timely management of sigmoid volvulus and help prevent adverse outcomes.

## Conclusions

Sigmoid volvulus can have severe consequences, such as intestinal ischemia and even mortality, if not addressed in a timely manner. Intestinal decompression can be preferred in patients with risk, but it is associated with recurrence, and hence, surgical correction is recommended, followed by intestinal preparation by decompression or other means. A successful outcome can be predicted with a prompt diagnosis and early treatment by a multidisciplinary approach, as noted in this case, where the sigmoid volvulus presentation was acute with no visible signs of edema or gangrene. A surgical management approach can be useful, as it is associated with lower recurrence rates in these patients than laparoscopic management.
